# Ozone and Semen Quality

**DOI:** 10.1289/ehp.115-a185b

**Published:** 2007-04

**Authors:** Jens Peter Bonde

**Affiliations:** Department of Occupational Medicine, Århus University Hospital, Århus, Denmark, E-mail: JPBON@as.aaa.dk

Sokol et al. (2006) reported an inverse association between environmental ozone and sperm concentration. They performed longitudinal analyses of > 5,000 semen samples from 48 semen donors over a 2-year period and concluded that exposure to average ambient O_3_ levels in the range of 20 ppb adversely effect semen quality.

Sokol et al. (2006) did not discuss available evidence on this issue from the occupational arena. Welding of metals with gas shielding of the weld, for example, tungsten inert gas (TIG) and metal inert gas (MIG) welding, confers an exposure to O_3_ that may reach a concentration of 400–600 ppb in the welder’s breathing zone (Korczynski 2000). In three cross-sectional semen studies and one longitudinal study, lower sperm counts were not reported among TIG and/or MIG welders compared with appropriate reference groups of nonwelding metal workers (Bonde 1990a, 1990b; Hjollund et al. 1998; Jelnes and Knudsen 1988).

The O_3_ exposure levels are some 20 times higher among the welders than among the residents in Los Angeles, California. Moreover, the environmental O_3_ measurements in Los Angeles performed outdoors, and indoor levels may be considerably lower. O_3_ is generated by ultraviolet radiation of oxygen and has a short half-life. Therefore, it can be assumed that the exposure of citizens is highly influenced by the time spent outdoors, which were not accounted for by Sokol et al. (2006). Could the weak associations they observed in their environmental study be artifacts of complex statistical analyses? In all circumstances, it seems too early to conclude that O_3_ alters semen quality.
